# A national scale coastal change dataset for Aotearoa New Zealand

**DOI:** 10.1016/j.dib.2024.111104

**Published:** 2024-11-03

**Authors:** Megan Tuck, Mark Dickson, Emma Ryan, Murray Ford, Teresa Konlechner

**Affiliations:** a The University of Auckland, Private Bag 92019, Auckland 1142, New Zealand; b The University of Otago, PO Box 56, Dunedin 9054, New Zealand

**Keywords:** Coastline, Historical aerial imagery, High-resolution satellite imagery, Coastal adaptation, Digital shoreline analysis system

## Abstract

Comprehensive historical coastal change datasets are crucial resources for effective coastal management. In Aotearoa New Zealand, available coastal change data is outdated, or sporadic, hindering large-scale, long-term analysis of coastal change, and coastal planning nationwide. Here we introduce New Zealand's Coastal Change Dataset (NZCCD) a detailed record of coastal change around New Zealand from the early 1940′s to 2023. NZCCD was generated through a nationally consistent, rigorous process in which five coastal scientists manually interpreted and digitized the position of the coastline along New Zealand's open and soft cliffed coasts, using historic aerial photographs and high-resolution satellite imagery. NZCCD provides two datasets i) *NZCCD Coastlines,* comprising manually mapped coastlines, and ii) *NZCCD Coastal Change Rates,* 228, 611 points where rates of coastal change were calculated. The dataset enables a significant advancement in coastal management enhancing our understanding of the patterns and drivers of coastal change around New Zealand.

Specifications Table

**Table:** 

Subject	*Environmental Science*
Specific subject area	*Coastal change, coastline mapping, coastal management and remote sensing*
Type of data	NZCCD *Coastlines,* Shapefile (.shp) , raw NZCCD *Coastal Change Rates,* Shapefile (.shp) , processed
Data collection	New Zealand's Coastal Change Dataset [1] is comprised of two datasets; *coastlines (*NZCCD Coastlines) and *rates (*NZCCD Coastal Change Rates) . The *coastlines* dataset was collected by manually mapping the New Zealand coastline using historical vertical aerial imagery and high-resolution satellite imagery within ArcPro 3.1.4. Open coast beaches and soft cliffed coasts were mapped. The *rates* dataset was generated using the Digital Shoreline Analysis System developed by the United States Geological Survey, calculating coastal change statistics from the *coastlines* dataset.
Data source location	*Country: New Zealand*
Data accessibility	Repository name: Figshare Data identification number: 10.17608/k6.auckland.27105955.v1 Direct URL to data: https://auckland.figshare.com/articles/dataset/New_Zealand_s_Coastal_Change_Dataset_NZCCD_/27105955 For improved data accessibility the dataset is mirrored on data.coastalchange.nz where users can visualize and sub-sample the data for specific interests and download the dataset in a variety of formats (.csv, .shp, .kml and .gpkg)
Related research article	*None*

## 1 Value of the Data

• This dataset is the first publicly available national collection of a) historic coastlines and b) rates of coastal change, for New Zealand.
• NZCCD provides an important baseline dataset on which to ground future projections of coastal erosion for adaptation decision-making and hazard planning. This standardized and robust national dataset provides the necessary data to inform models projecting the future position of the coastline.
• Researchers and practitioners can utilise New Zealand's extensive coastal change dataset to explore the modes, styles and patterns of coastal change across New Zealand's diverse coastal environments including sandy and gravel beaches, cliffed coasts, and active dunes.
• A comprehensive coastal change dataset is crucial for understanding the drivers of coastal change and their interaction with SLR. NZCCD provides the necessary data for researchers to explore the drivers of coastal change in New Zealand and provides a baseline against which to evaluate future changes. NZCCD also provides the necessary data to explore the influence of various drivers on coastal change in New Zealand.
• A wide range of stakeholders might utilise this data including coastal hazard managers and planners who may use the data to develop and improve coastal hazard management strategies such as setback lines and rolling easements. Furthermore, decision-makers can incorporate analysis of the coastal change dataset into adaptation planning tools, such as Dynamic Adaptive Policy Pathways (DAPP), to improve community coastal adaptation planning processes [2,3].
• This dataset is also valuable to communities and individuals in New Zealand who can use NZCCD to gain detailed knowledge on coastal erosion risk at their local beaches.

## 2 Background

Accelerating rates of sea level rise threaten coastal communities globally, yet our understanding of the impact of rising sea levels on the coast is not well understood, in part, due to a limited understanding of historical coastal change [[4], [5], [6]]. Comprehensive historical coastal change datasets, that map the position of the coastal boundary over time, are necessary for informed decision-making and for developing effective adaptation strategies to manage and mitigate the impacts of sea level rise on coastal regions. Despite this, New Zealand, a coastal nation with 65 % of people living and working near the coast [7], lacks an up-to-date national coastal change assessment.

The last large-scale assessment of coastal change in New Zealand was conducted by Gibb [8] in 1978, who measured changes in the position of the coast at 310 locations, providing a total of 471 measurements of coastal change around New Zealand, but lacking detail on local-scale variability [8]. Since Gibb's study, coastal change assessments in New Zealand have been sporadic, typically undertaken only at individual beaches where erosion concerns are paramount and have employed differing methodologies [[9], [10], [11]]. The ad hoc nature of existing coastal change assessments in New Zealand hinders comparisons and cross-project usage of data. Data compilation and standardisation across coastal regions is required to explore large-scale, long-term analysis of coastal change, and support rigorous coastal planning nationwide.

## 3 Data Description

*NZCCD* [1] is comprised of two datasets; *coastlines ^(^*NZCCD Coastlines) and *rates (*NZCCD Coastal Change Rates) datasets. The *coastlines* dataset comprises all New Zealand's mapped coastal positions from the 1940′s to 2023, covering over 3000 km of New Zealand's open coast. The *rates* dataset comprises coastal change statistics at 10 m intervals along the mapped coastline. NZCCD consists of 2676 unique coastlines, totalling 19, 449 km of coastline interpreted and mapped from remote imagery, and 228, 611 points along the coast where coastal change statistics have been calculated. The dataset is stored in a vector format in the NZGD 2000 New Zealand Transverse Mercator coordinate system. The dataset can be freely accessed through Figshare [1] or data.coastalchange.co.nz where users can draw a box around the area they would like to download data for and download the data in a variety of formats including shapefile (.shp), Keyhole Markup Language (.kml) and Geopackage (.gpkg).

## 4 Experimental Design, Materials and Methods

### 4.1 Data acquisition

#### 4.1.1 Source imagery

New Zealand's coastlines were digitised manually within ArcPro from historical vertical aerial imagery (available for New Zealand since ∼1940) and very high-resolution satellite imagery (available from early 2000s). Aerial and satellite imagery offer the only nationally consistent datasets suitable for mapping coastal change in New Zealand, providing high-resolution imagery with extensive coastal coverage.

#### 4.1.2 Sourcing vertical aerial photographs

Vertical aerial photographs have been essential for mapping coastal change across a range of temporal scales and spatial settings [12,13]. Many developed nations have temporally rich collections of aerial imagery covering their coastline, providing abundant data for coastal change assessments. The earliest period of aerial imagery available in Aoteraoa New Zealand is from the late 1930′s. Subsequent to this, periodic regional surveys were undertaken up until the early 2000′s, but survey frequency has increased since 2010 after which regional aerial surveys are flown every few years. Historic aerial imagery for New Zealand was accessed from New Zealand's Crown Archive from Retrolens (Retrolens.co.nz) who provide unprocessed scans of aerial imagery, and from the Land Information New Zealand Data Service (data.linz.govt.nz) who provide published orthophotography. All available vertical aerial imagery covering New Zealand's coast was downloaded for this project, most sections of the coast have imagery extending back to the 1940′s, however the imagery record at a few locations starts in the 1950′s or 1960′s.

#### 4.1.3 Sourcing high-resolution satellite imagery

For the last twenty years, very high-resolution (VHR) optical satellite imagery has been increasingly utilised for coastal change assessments [14,15]. This type of imagery offers enhanced accuracy in mapping coastlines and allows for more frequent analyses than is possible with aerial imagery, enabling the examination of coastal behaviour over various temporal scales. VHR satellites typically capture both multispectral images; which include bands of blue, green, and red light, as well as near-infrared and short-wave infrared, and panchromatic images; a single band that captures a broader portion of the visible spectrum at a higher spatial resolution than the multispectral bands. Pan-sharpened images, generated by fusing the higher resolution panchromatic band with the lower-resolution multispectral bands, provides high-resolution (typically between 30 and 50 cm) multispectral imagery for accurate coastal mapping. VHR satellite imagery is available from various commercial companies, such as Maxar which operates VHR optical Earth observation satellites with archives dating back to the early 2000s for most of New Zealand. Satellite imagery was typically purchased from existing imagery archives. However, when commercial satellite imagery was not available for specific dates of interest, imagery was tasked by specifying criteria for location and date range for new image captures.

#### 4.1.4 Georeferencing imagery

The georeferencing process involves correctly placing an unregistered aerial or satellite image in a chosen geographic projection by matching ground control points (GCPs) between the unregistered image and a source of ground control. In this case, high resolution ortho-rectified mosaics from Land Information New Zealand (LINZ) were used as the source of ground control.

**Fig. 1:**
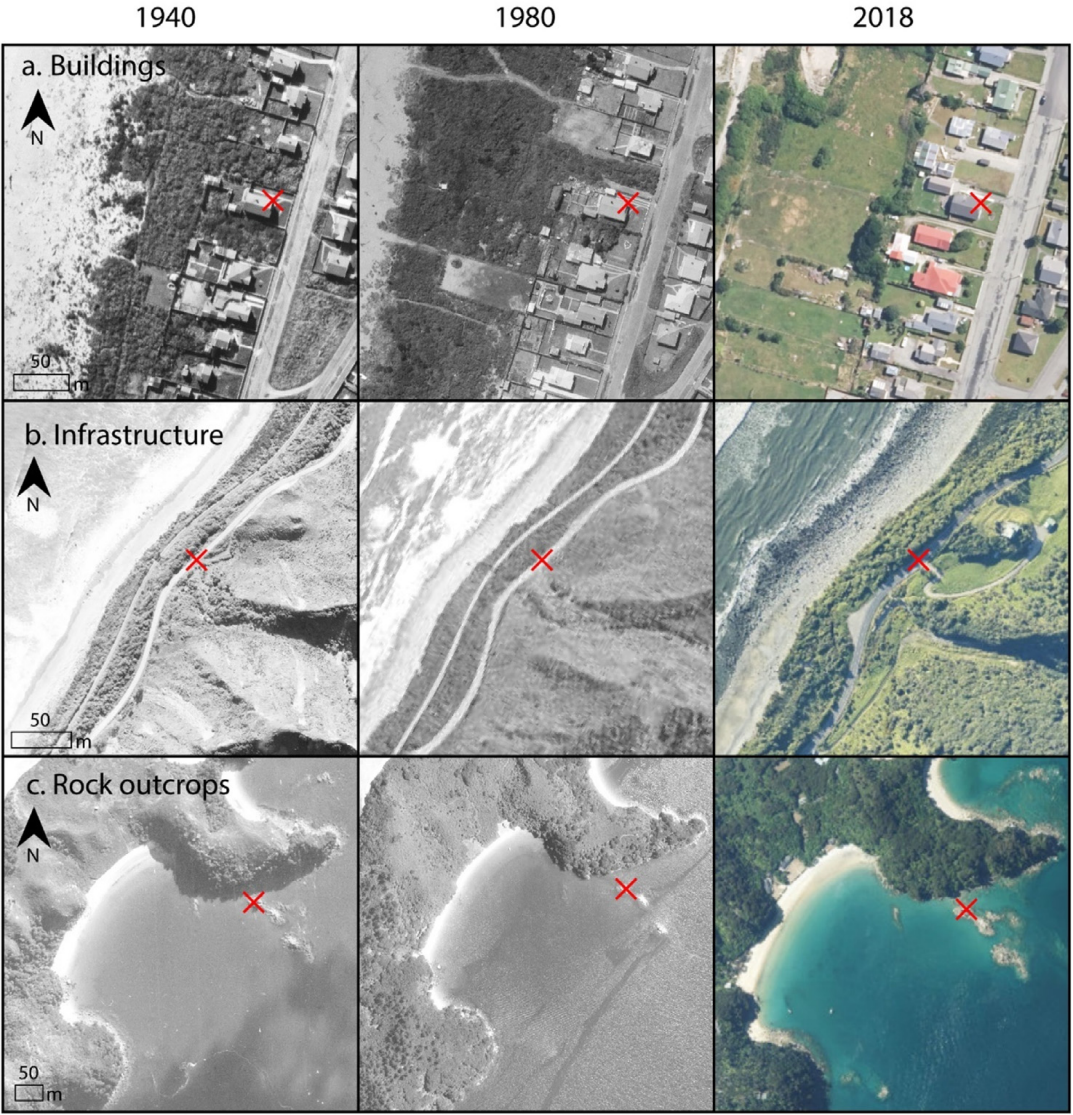
Examples of a) and b) anthropogenic, and c) natural, features used as reliable ground control points for georeferencing aerial and satellite imagery at the coast in New Zealand.

Georeferencing historical aerial imagery within coastal settings is often challenging due to a lack of reliable ground control points. Anthropogenic features such as houses or roads, or stable geologic features such as large rocks, are often absent in sandy sections of the coast and particularly in non-urban areas. When georeferencing coastal imagery for New Zealand a mix of natural and anthropogenic features were used as GCPs. Anthropogenic features that had not been moved or modified through time, such as roads, bridges, buildings and fence lines, were used where possible. In some cases, stable natural features such as rocky outcrops were the most reliable GCPs that could be used to position the image (Fig. 1). Fig. 1 illustrates an example of a building, road and rocky outcrop in the same position in 1940, 1980 and 2018. The number of GCPs used for each image varied between 12 and 20 and were concentrated along the coast the ensure the image was well georeferenced along the coastline. In areas where both reliable anthropogenic and natural features were absent, often in remote sandy landscapes, Abobe Photoshop was used to mosaic adjacent images to create a single larger image. Once mosaiced, reliable GCP's from the neighbouring imagery could be used to place the mosaiced image in the correct geographic location.

**Fig. 2:**
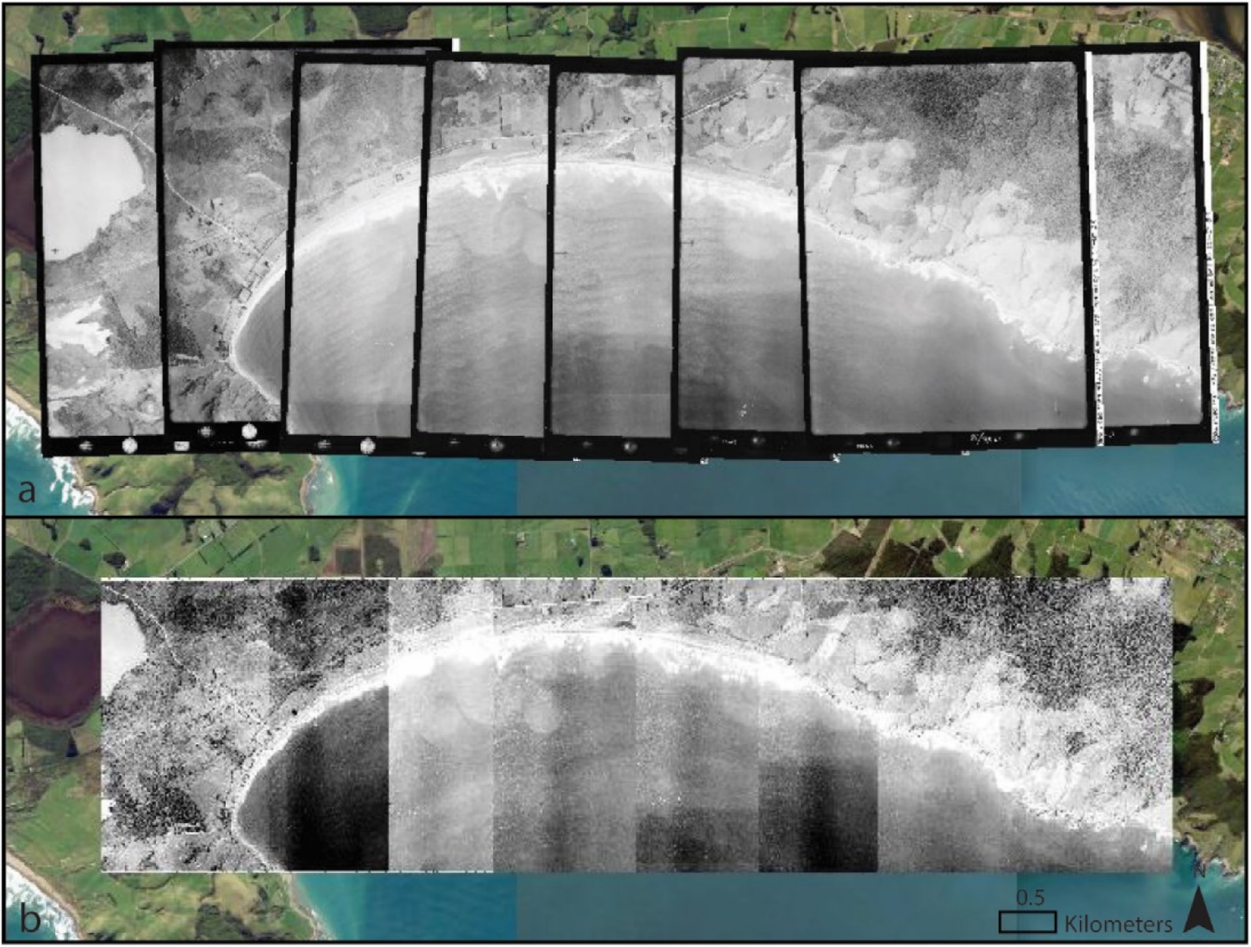
a) Georeferenced and b) mosaiced historic aerial images over high-resolution satellite imagery of Colac Bay, Southland, New Zealand.

Image orthorectification was not undertaken due to due to budget and data availability constraints. To avoid topographic distortion, GCPs were concentrated along the coast at approximately the same elevation. Unorthorectified images have been reliably used for coastal mapping in a wide range of coastal settings [9,16]. Once georeferenced, neighbouring historical aerial images along a stretch of coast were mosaiced removing image borders to aid the coastline mapping process. All georeferencing and mosaicing was undertaken in ArcPro 3.1.4 [17]. Georeferenced (a) and mosaiced (b) historical aerial imagery from 1952 at Colac Bay, Southland is shown in Fig. 2.

#### 4.1.5 Interpreting and digitising the position of the coastline

The position of the coastline is often interpreted as the boundary between sand and water, however, multiple indicators or proxies might be used to record coastline position in different coastal environments [18]. When selecting a proxy to capture the position of the coast, it is important to consider the timescale over which coastal change is being investigated, as well as the ability to interpret the chosen proxy within the available aerial and satellite imagery. Multi-decadal coastal change studies [9,13] often use the edge of vegetation (EOV) as a coastal change proxy as it is expected to reflect longer-term erosion and accretion patterns compared to the instantaneous water line (IWL) or high-water line (HWL) , which are sensitive to changes in wave run up, tides, and storm surge [18]. The EOV is also readily identifiable within all sources of imagery regardless of image colour and contrast and during most environmental conditions. In contrast, the IWL and HWL can be difficult to identify in instances where waves and glare can impede their interpretation and they can be mistaken for other coastal features, especially in low-quality scanned black-and-white photographs [18].

**Fig. 3:**
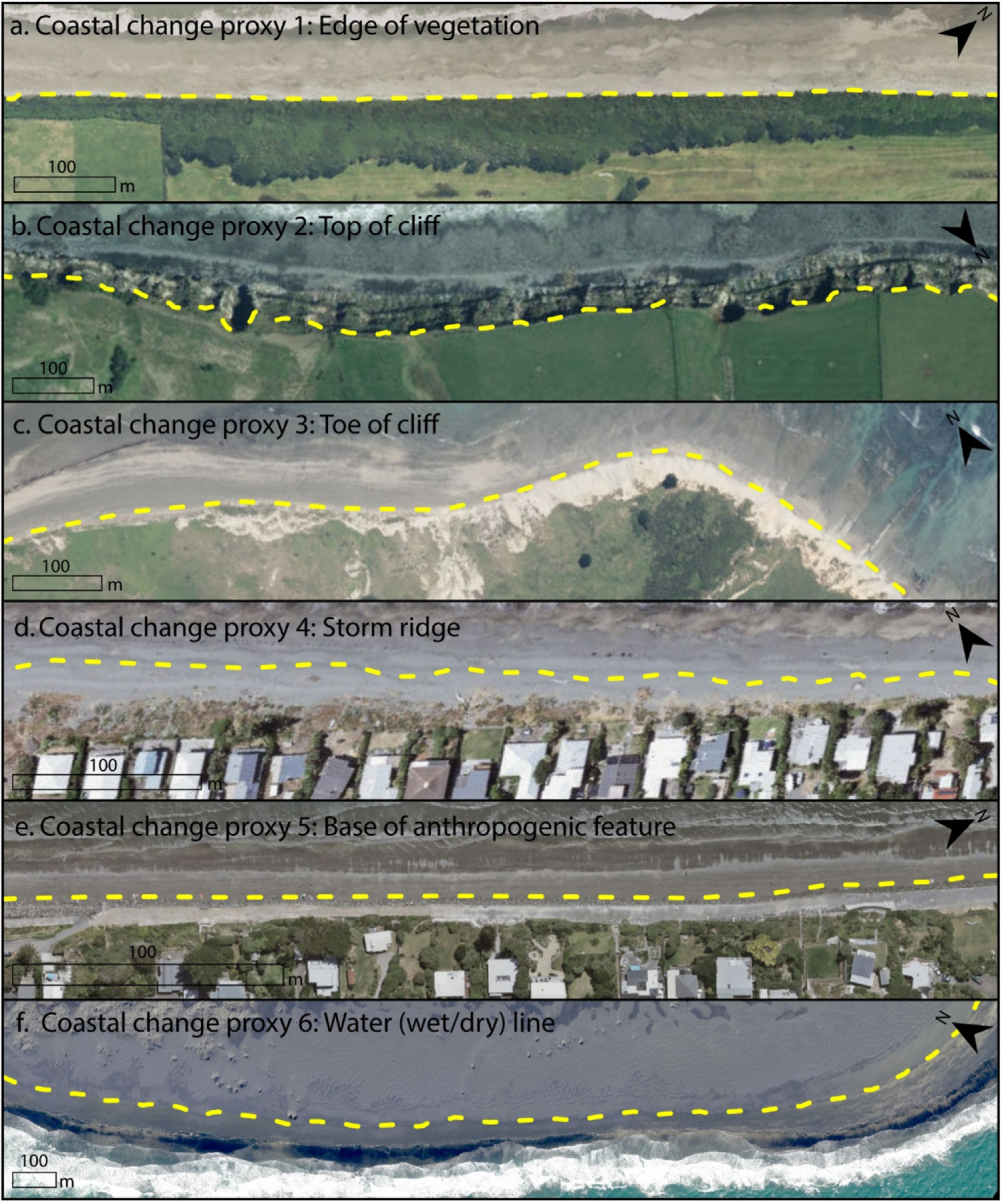
Coastal change proxies used to record coastal change for New Zealand's Coastal Change Dataset. 1. Vegetation line was used for most sandy coasts. 2. Top of cliff or 3. Toe of cliff were used to map cliffed coasts. 4. Gravel barrier or storm berm were used to map gravel beaches. 5. Anthropogenic features were used to map coasts defended by sea walls and revetements. 6 Wet/dry line was used to map coastal change when no other proxy was appropriate or visible within all images.

New Zealand is characterised by dynamic coastal environments including both sandy and gravel beaches, a variety of cliff geologies, and active coastal dunes. Considering the variability of New Zealand's coastline, this study adopts a variety of coastal change proxies, each easily identifiable within all aerial and satellite imagery used (Fig. 3). For each coastal environment mapped the most appropriate proxy was chosen and kept consistent for every image digitised at that location. The edge of vegetation was chosen as the proxy for the majority of New Zealand's coastline (77 %) and was mapped for all sandy beaches backed by a dune, while the gravel ridge (often corresponding to the storm ridge) was used to represent the coastline for gravel beaches (7 %). Either the cliff top (7 %) or cliff toe (6 %) was mapped for cliffed coasts (depending on the height and nature of the cliffs) , and the water wet/dry line was used on scarce occasions when no other proxy was applicable (2 %) . Where coasts are defended by sea walls or rock revetments, the base of the anthropogenic feature was mapped (1 %) (Fig. 3).

New Zealand was divided into ∼430 Areas of interest (AOIs) about 5–10 km in length and comprised all open coast beaches and soft cliffed coasts around New Zealand. Hard-rock cliffs, which have imperceptible erosion rates over the historic period, were not mapped. For consistency, coastal mapping at each AOI was conducted by a single experienced operator using ArcPro 3.1.4 [17], and at a uniform scale (1:1000 - 1:2000) based on image resolution. New Zealand coastlines were digitised and saved as vector polyline shapefiles. Metadata were captured and stored within the shapefiles (Table 1).

**Table 1:** New Zealand's Coastal Change Dataset: NZCCD Coastlines attribute table and fields explained.

Attribute	Example	Description
Region	Southland	The region in New Zealand that the coastline is from.
Date	10/04/1955	Coastline date in DD/MM/YYYY format
Source	RL	The type of imagery used to digitise the coast. RL = Retrolens, VHR = satellite imagery, LDS = Linz Data Service
Proxy	1	The proxy value denotes the type of coastal change proxy digitised e.g. edge of vegetation (1) or storm ridge (4), see Fig. 3.
Total_UNCY	2.3	The uncertainty associated with the segment of the coastline. Total uncertainty is calculated from the georeferencing error, pixel error and digitiser error (see Technical Validation).

#### 4.1.6 Calculating rates of coastal change

**Fig. 4:**
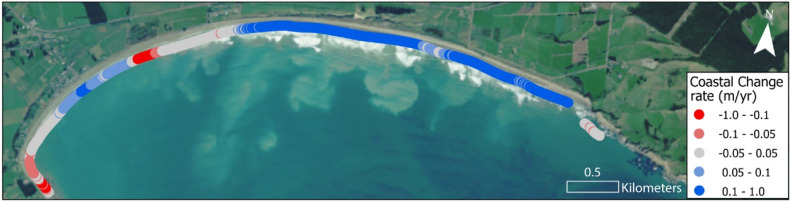
Rate of coastal change at Colac Bay, Southland between 1953 and 2023.

Robust timeseries of coastline positions can be used to calculate rates of coastal change and provide an important baseline dataset on which to ground future projections of coastal erosion for adaptation decision-making and hazard planning. The Digital Shoreline Analysis System (DSAS) developed by the United States Geological Survey (USGS) was used to generate transects at 10 m intervals along the coast. DSAS was used to analyse and report coastal change statistics for each transect [19] (Fig. 4, Table 2).

**Table 2:** New Zealand's Coastal Change Dataset: NZCCD Coastal Change Rates attribute table and fields explained.

Attribute	Example	Description
Region	Northland	The region in New Zealand that the coastline is from.
Start_Date	21/03/1943	The date of the earliest coastline used in analysis in DD/MM/YYYY format
End_Date	05/11/2022	The date of the latest coastline used in analysis in DD/MM/YYYY format
Duration	77	The duration (rounded to nearest year) of the coastline record at this point.
ShrCount	7	The number of coastlines used in analysis
Net Shoreline Movement	NSM	The distance (m) between the earliest and the latest coastline for each transect.
Shoreline Change Envelope	SCE	The distance (m) between the most landward and most seaward coastline.
End Point Rate	EPR	The rate of change (m/year) between the oldest and most recent coastal positions is calculated by dividing the distance by the time between the two coastlines.
End Point Rate Uncertainty	EPRunc	Uncertainty of the end point rate (m).
Linear Regression Rate	LRR	The linear regression rate of change is determined by fitting a least-square regression line to all coastline points for a transect.
Confidence Interval	LCI	The confidence interval of linear regression
Standard Error	LSE	Standard error of linear regression
R-Squared	LR2	The LRR R-squared statistic is a dimensionless index (ranging from 1.0 to 0.0) that describes the scatter (variance in the data).
Weighted Linear Regression Rate	WLR	The more reliable data (lower uncertainty) are given a greater weighting in a WLR.
Confidence Interval	WCI	Confidence interval of weighted linear regression
Standard Error	WSE	Standard error of weighted linear regression
R-Squared	WR2	The WLR R-squared statistic is a dimensionless index (ranging from 1.0 to 0.0) that describes the scatter (variance in the data).

#### 4.1.7 Technical validation

A rigorous uncertainty assessment was undertaken to calculate the positional uncertainty of the coastlines mapped from remote imagery for NZCCD. Total shoreline uncertainty (*E_t_*) is calculated as the root sum of squares of the pixel error (*E_p_*) , georeferencing error (*E_g_*) and digitising error (*E_d_*).

(1) $$E_{t}=\sqrt{E_{p}^{2}+E_{g}^{2}+E_{d}^{2}}$$

The pixel error (*E_p_*) is the resolution of the georeferenced mosaic from which the coast is mapped. The georeferencing error (*E_g_*) is the 95 % confidence interval of the spatial accuracy of the imagery. This is determined through independent checking of the positional accuracy of the mosaics. A digitiser error (*E_d_*) is adopted based on the characteristics of the coastline being interpreted along with testing of the ability of the digitiser to interpret and digitise the same feature [20].

Total shoreline uncertainty (*E_t_*) is represented by Total_UNCY in the attribute table of the coastlines (*NZCCD_coastlines)* dataset (Table 1). Total shoreline uncertainty ranged between 0.4 m and 10.9 m with the highest uncertainty associated with low resolution, black-and-white historical imagery while VHR satellite imagery had the lowest shoreline uncertainty. The average shoreline uncertainty is 4.15 m. Please see Ford et al. [20] for further details of the uncertainty assessment followed to assess the accuracy of the coastal positions mapped from remote imagery.

## Limitations

The collection of the dataset was constrained by the variable availability of historic aerial imagery covering New Zealand's coastline. Historic aerial imagery is more abundant along more densely populated coasts, leading to inconsistencies in the temporal coverage of coastal change data throughout New Zealand. For sections of the coast with a rich historical imagery record, typically near urban areas, the dataset extends back to the early 1940′s. In contrast, for some rural and less populated coasts, the imagery record often begins later, in the 1950′s or 1960′s, due to a limited historical imagery record. This variability is documented in the NZCCD attribute table, which includes fields for the ‘Start_date’, ‘End_date’ and ‘Duration’ of the coastal change record at each point where coastal change statistics are calculated.

Time and budget constraints also impacted the scope of the dataset where only open and soft cliffed coasts were mapped, omitting harbours and inlets. Additionally, not all of New Zealand's open coast beaches were mapped and included in NZCCD. However, we aim to continue updating NZCCD to eventually provide coastal change data for the entirety of New Zealand's open coast.

## Ethics Statement

The dataset does not involve studies with humans, animals or data collected from social media platforms. We confirm that this research strictly adheres to the ethical requirements for publication provided by Data in Brief.

## Credit Author Statement

**Megan Tuck:** Methodology, Formal analysis, Investigation, Data Curation, Writing – Original Draft, Visualisation. **Mark Dickson:** Conceptualisation, Methodology, Formal analysis, Investigation, Writing – Review & Editing, Funding acquisition. **Emma Ryan:** Conceptualisation, Methodology, Formal analysis, Investigation, Writing – Review & Editing. **Murray Ford:** Conceptualisation, Methodology, Formal analysis, Investigation, Writing – Review & Editing. **Teresa Konlechner:** Investigation, Writing – Review & Editing.

## Data Availability

FigshareNew Zealand's Coastal Change Dataset (Original data).
